# Association of IL-17A Polymorphism with Chronic Periodontitis in Type 1 Diabetic Patients

**DOI:** 10.30476/DENTJODS.2020.86990.1222

**Published:** 2021-09

**Authors:** Sasi Kumar PK, Sheeja Varghese S, Thanga Kumaran, Jaga Desaan N, Lambo Daran G

**Affiliations:** 1 Dept. of Periodontics, JKKN Dental College, Erode, Tamilnadu-638183, India, Ph.D scholar, Saveetha Institute of Medical and Technical Science, Chennai, Tamilnadu; 2 Dean, Saveetha Dental College, Saveetha Institute of Medical and Technical Science, Chennai, Tamilnadu, India; 3 Dept. of Periodontics, JKKN Dental College, Tamilnadu, India; 4 Dept. of Prosthodontics, JKKN Dental College and Hospital, Tamilnadu, India; 5 Dept. of Prosthodontics, Meenakshiammal Dental College, Chennai, Tamilnadu, India

**Keywords:** IL-17A, Polymorphisms Type 1 Diabetes, Chronic Periodontitis, Ethnicity

## Abstract

**Statement of the Problem:**

The association of genetic polymorphisms with periodontitis has been studied extensively. The interleukin -17 (IL-17) is a group of cytokines, which comprises six different molecules (IL-17A, B, C, D, E & F). Among these, IL-17A & F are the most commonly understood cytokine, which plays a critical role in inflammatory diseases and periodontal inflammation.

**Purpose:**

To evaluate whether IL-17A gene polymorphism is associated with increased risk of chronic periodontitis in type 1 diabetes patients.

**Materials and Method:**

This quantitative case- control study was carried out in 60 subjects in 4 groups. The study groups included group A: 15 type 1 diabetes patients (T1DM) with chronic periodontitis (CP), group B: 15 T1DM patients without CP, group C: 15 Non-diabetic patients with CP, group D: 15 Non-diabetic patients without CP. Blood samples were drawn from the subjects and analyzed for IL-17A polymorphism by using the polymerase chain reaction-restriction fragment length polymorphism method.

**Results:**

There was no statistical significant difference seen in the genotype distribution among CP patients with or without T1DM and healthy controls. Odds ratio and p value indicated that increased risks for CP were associated with IL-17A allele (G) in patients with T1DM. This allele was correlated with worse clinical parameters of CP in T1DM patients.

**Conclusion:**

The present study revealed that IL-17A (rs2275913) polymorphism was not associated with increased risk for CP in T1DM patients.

## Introduction

Chronic Periodontitis is a complex infectious- inflammatory disease, immune response play a major role in irreversible destructions of gingival tissue, periodontal ligament, and alveolar bone destruction [ 1 ]. The periodontal pathogens initiate the activation of innate and adaptive immunities, which leads to the release of inflammatory mediators from host tissues [ 2 ]. This excessive production of inflammatory mediators triggers the tissue destruction and provokes further progression of the disease. This progression can be attributed to the more production of pro-inflammatory cytokines and decreased amount of anti-inflammatory mediators [ 3 ]. The main factors in the immunoregulation of periodontal disease depend on the controlled balance between the T helper 1 & 2 (Th 1/Th2) cells [ 4 ]. 

Among the T-helper cells, Th17 subset is recently established prominent focus, because of its role in the autoimmune disease pathogenesis. The interleukin -17 (IL-17) consists of six groups of cytokines (IL-17A to IL-17F) and is expressed by the CD4^+^ T-helper subset (T-helper 17 lineage) and mediates the tissue inflammation [ 5 ]. This causes the activation of T cells, fibroblast-s, osteoclast, and the maturation of dendritic cells. Rep-orts indicate that IL-17A has a key role in the progression of chronic and aggressive periodontitis [ 6 - 7 ]. IL-17A triggers the proinflammatory cytokines such as TNF-α, IL-6, and IL-1β expressions and promotes neutrophil migration. It can facilitate the function of CD 8^+^ T cells and causes the B cell activation to produce antibodies. These indicated that IL-17A cytokines play a role in potentially promoting inflammatory and periodontal disease [ 8 ].

IL-17 cytokines are also involved in the initiation of autoimmunity in rheumatoid arthritis, experimental autoimmune encephalomyelitis, multiple sclerosis, systemic lupus erythematosus, and auto-immune diabetes (T1DM) [ 9 - 12 ]. The risks of T1DM are ALA-associated genotypes and B cell autoantibodies. Recently, the role of T helper 17 (Th 17) cells, which secrete IL-17 in pathogenicity, was also suggested as a risk factor in T1DM development [ 13 ]. IL-17 gene is located on each side of the human chromosome 6p12.2. IL-17 shares 50% amino acid with chromosome 6 and similar functions (almost 50%) as that of chromosome 6. In vitro study revealed that in diabetic patients, there was increased expression of IL-17A, which was primarily induced by monocytes [ 14 ]. IL-17 producing cells were commonly found in peripheral blood and among the monocytes of chronic T1DM .The activation of this IL-17 pathway accelerates the apoptosis of pancreatic β cells, leading to T1DM [ 15 ]. The studies done on two different groups of children with new onset of T1DM revealed that there was increase in number of IL-17 producing T-cells in their peripheral blood [ 16 ].

IL-17A cytokine in periodontal diseases was significant associated with disease progression and severity of destruction. IL-17A levels were also significantly elevated in saliva and gingival crevicular fluids of patients with chronic periodontitis [ 17 - 18 ]. IL-17A polymorphism was studied in the literature; it was found to be commonly associated with diabetes, and chronic and aggressive type of periodontal disease [ 19 - 20 ]. The single nucleotide polymorphisms (SNP) in the lL-17 gene had important effect on the production of IL-17 in T1DM. In addition, Linhartova *et al*. [ 19 ] showed that the correlation between increased levels of IL-17 and A allele of 197A/G SNP16. The role of this A allele was also marginally associated with the increased risk of T1DM and CP. The mechanism of IL-17 in human T1DM provides a new view on the pathogenesis of the disease with periodontitis and implies a novel potential therapeutic strategy in T1DM by controlling the role of IL-17. There were many studies showing a contradictory role of Th17 in both protection and pathogenesis of T1DM and CP [ 17 - 20 ]. Hence, this study was to done to evaluate the association of IL- I 7A polymorphism in CP with T1DM patients.

## Materials and Method

The study was taken out in the outpatient Department of Periodontics, JKKN Dental College and Hospital. The ethical form was received after approval from the Institutional Committees for Ethics and informed written consent was taken from all participants before their participation in the study. The study was carried out in 60 subjects in four groups. Group A consisted of 15 T1DM patients with CP, group B included 15 T1DM patients without CP, group C consisted of 15 non-diabetic patients with CP, and group D included 15 non-diabetic patients without CP. 

Inclusion criteria for T1DM were both male or female patients, age group between 30-44 years, those who have already diagnosed with T1DM, both controlled and uncontrolled level of glycemic [Glycemic index (HbA1c) was taken for grouping] patients. Inclusion criteria for CP were cases diagnosed based on AAP criteria stage-II for generalized chronic periodontitis, which includes presence of clinical attachment loss 3-4mm, pocket probing depth (PPD) of 5mm at least more than 30% teeth involved, and 15% to 30% coronal third radiographic bone loss [ 21 ]. Exclusion criteria were smokers, ongoing orthodontic therapy, aggressive periodontitis, general health problems (hepatitis, HIV infection, and chemotherapy), pregnancy, lactation, and non-Indian races. Periodontal parameters were measured using Williams probe and the gingival Index was measured for assessing the severity of gingivitis [ 22 ]. Diabetic parameters were monitored with HbAlc and fasting blood glucose levels (FBS). HbA1c value of <7% revealed there was sufficiently good control of blood glucose in T1DM patients and FBS was monitored with cutoff points of > 120mg/dl [ 23 ].

### DNA Extraction

The venous blood (5ml) was collected from cubital fossa of the patients under strict sterile conditions and transferred to the laboratory in a falcon tube containing ethylenediaminetetraacetic acid (EDTA) [ 16 ]. It was stored at -200^o^C for DNA separation. The 200μL of the blood sample was placed into a micro centrifuge tube and 600μL of RBC cell lysis solution was added to the blood sample. The uniformity in the sample mix was obtained by inverting the tube several times and flicking the bottom of the tube. The incubation of the sample was done for 5 minutes at room temperature and shaken briefly. Again, it was repeated at room temperature for 5 minutes, followed by brief agitation of the tube. The centrifuging was done in 25 seconds with 4000 rpm at 4^o^C. The supernatant was removed, leaving approximately 25μL of liquid in the tube. Then, 300 μL of tissue and cell lysis solution was added and by pipetting the cells several times. Then 1μL RNase A solution added, thoroughly mixed, and incubated for 30 minutes at 37^o^C. The samples were cooled by placing on the ice for 3 to 5 minutes. Then, MPC protein precipitation reagent of 150μL of was added to 300μL of the list sample. The test tube was again agitated briefly. The centrifugation was done in 4^o^C for 10 minutes at 10,000 rpm. If the resultant pellet is clear or loose, MPC protein precipitation reagent mix of 25μL is added and then debris should be removed. The debris was discarded and supernatant was transferred to a clean micro centrifuge tube. Then 500μL of isopropanal was added to the recovered supernatant. The tube was inverted for 30-40 times. Then keep it for centrifugation at 4^o^C for 10 minutes. Then carefully pace of the isopropanol from micro centrifuge tube without dislodging the DNA pellet. The final pellet was rinsed twice with 70 percent ethanol without dislodging. If the pellet was dislodged, centrifuging was done briefly. Finally, all the residual ethanol was removed with the pipette and DNA was resuspended in 35μL of TE buffer.

### DNA Amplification by Polymerase Chain Reaction

The primer pair (Bioserve, Beltsville, USA) used for this study to check IL- l7A polymorphism was, Sense: IL-17AF

5’-AACAAGTAAGAATGAAAAGAGGACATGGT-3’

Antisense: IL-17AR

5’-CCCCCAATGAGGTCATAGAAGAATC-3’

The amplification was done using conventional PCR system with a cycle consisting of

i) A denaturation step at 950 C for 15 minutes initially,

ii) Then, denaturation step of 35 cycles for 30 s at 940 C.

iii) Annealing was performed for 35 cycles for 30 s at 57^o^C. 

iv) Then, the extension step was about 35 cycles at 720 C for 30 s 

iv) Final extension step at for 10 minutes 72^o^C. 

The incubation of PCR products was done overnight with Xagl (Waltham, USA) at 37^o^C. The 6.5% polyacrylamide agarose gel electrophoresis system stained with silver was used for viewing the bands.

The real-time PCR amplification was used for determining the IL-17A (rs2275913) alleles and the typing results was analyzed by using a Roche Light Cycler 480 instrument. The detection of IL-17A polymorphism was done using LightSNiP (rs2275913) assay developed by TlB MOLBlOL (GmbH, Berlin, Germany).

IL-I 7A genotype distribution and allelic variations in the groups were measured by Chi-Square test. The multiple comparisons (Bonferroni correction) test was used to compare the periodontal parameters between the groups (Mean±SD). The odds ratio (OR) calculation was done using a 95% of confidence intervals (CI).

## Results

In total, 60 cases (35 females, 25 males) aged between 30-44 years were included in the present study. The comparisons between groups as well as periodontal status are given in Table 1. We evaluated the frequency of dominant (A) and recessive (G) allele to determine Hardy-Weinberg equilibrium (HW). The frequency of allele (A^2^, G2) was worked out to be 0.61, 0.39 respectively. The distribution of IL-17A polymorphism (A+G= 1) in each group was within HW. The comparison of three genotypes (AA, AG, and GG) was done in T1DM with CP, only CP and healthy patients. The genotype distributions of the IL-17A polymorphisms in each group are shown in Table 2. In this, IL-17A polymorphism showed no statistical significant difference in genotype distribution among the study groups (Control vs. Diabetes with CP and Control vs. Chronic Periodontitis). No association was found between IL-17A polymorphism in CP, T1DM, and healthy patients (*p*> 0.05). The differences in genotype frequency between patients and control (Group D vs. Group A, Group D vs. Group B, Group D vs. Group C) were not statistically significant. Hence, genotype frequencies were not statistically significant between healthy and diseased patients; GG genotype was shared higher percentage (73.3%, 66.6%, and 46.6%) of distribution among all the diseased groups. 

**Table 1:** Comparing the mean level of clinical periodontal parameters (gingival Index, periodontal probing depth, and clinical attachment loss) in chronic periodontitis with type 1 diabetes patients

Variable	Group	Mean±SD	F	Multiple comparison (Bonferroni correction)			
				Healthy vs. TIDM+CP	Healthy vs. TIDM	Healthy vs. CP	TIDM+CP vs. TIDM
GI	TIDM+CP	1.93±032	198.76	*p*< 0.01*		*p*< 0.01*	
	T1DM	0.81±0.32			1.02		0.97
	CP	1.89±0.54			*p*> 0.01		*p*> 0.01
	Healthy	0.23 ±0.34					
PPD (mm)	TIDM+CP	4.09 ± 0.98	98.54	*p*< 0.01*		*p*< 0.01*	
	T1DM	2.97 ± 0.56			0.48		0.31
	CP	4.85 ± 0.34			*p*> 0.01		*p*> 0.01
	Healthy	2. 92 ±0.45					
CAL (mm)	TIDM+CP	2.86 ±0.74	87.62	*p*<0.01*		*p*< 0.01*	
	T1DM	0.69 ±0.54			0.62		0.53
	CP	3.06 ± 0.60			*p*> 0.01		*p*> 0.01
	Healthy	0.79 ± 0.02					

* *p*<0.05 then it was considered significant.GI –Gingival index, PPD-Probing pocket depth, CAL-Clinical attachment loss, CP-Chronic Periodontitis, T1DM-Type 1 diabetes, SD-Standard deviation.

**Table 2:** Distribution of the IL-17A genotypes in the groups (Chi-square test)

Variable	Group A		Group B		Group C		Group D		Healthy vs. TIDM+CP(p)	Healthy vs. TIDM(p)	Healthy vs. CP(p)	TIDM+CP vs. TIDM(p)
	n=5	%	n=15	%	n=15	%	n=15	%				
AA	0	0	0	0	2	13.3%	1	6.6%	-	-	0.006	-
AG	4	26.6%	5	33.3%	6	40%	5	33.3%	0.0065	0.0144	0.0071	0.034
GG	11	73.3%	10	66.6%	7	46.6%	9	60 %	0.0311	0.0305	0.0328	0.007
AA+AG	4	26.6%	5	33.3%	8	53.3%	6	39.9%				
AA+AG vs. GG	4 vs. 11	26.6% vs. 73.35%	5 vs. 10	33.3% vs. 66.6%	8 vs. 7	53.3%vs 46.6%	6 vs. 9	39.9%vs 60%	0.068	0.0174	0.120	0.023
H-W	0.5421 (p)		0.3642 (p)		0.1476 (p)		0.9652 (p)					

*p*< 0.05 significance for H-W. *p*> 0.05 for chi-square test. H-W: Hardy-Weinberg equilibrium, Group A: T1DM+CP, Group B: T1DM without CP, Group C: Chronic Periodontitis (CP), Group D: Healthy Group, T1DM: Type 1 diabetes mellitus

The dominant (AA, AG) and recessive genotype (GG) models in each group we,re analyzed and compared with the healthy group (group D). T1DM with CP (Group A) had a statistical significant difference when compared (AA+AG vs. GG) with a healthy group (*p*= 0.068) (Table 2). When considering the OR, 95% CI, T1DM+CP group had no association of IL-17A polymorphism, even though *p* Value was significant (OR=l .021 CI (0.654-3.543) ( Table 3).

**Table 3:** Distribution of genotypes (AA, AG and GG), alleles (A, G) in the groups

Variable	Group D vs. A OR (95%CI)	Group D vs. B OR (95%CI)	Group D vs. C OR (95%CI)	Group A vs. B OR (95%CI)
AA	--	---	0.567(0.435-1.0341)	
AG	0.345(0.087-1.135)	0.765(0.543-2.987)	0.432(0.308-1.047)	0.876(0.765-2.098)
GG	1.043(0.895-2.065)	0.654(0.611-1.0547)	1.065(0.871-1.913)	0.743(0.691-2.650)
AA/AG vs. GG	1.021 (0.654-3.543)	0. 802( 0.634-1.002)	1.207(0.765-2.755)	0.987(0.801-1.093)
A	0.732 (0.314-1.986)	0.765 (0.396-1.743)	0.901(0.864-1.491)	0.763 (0.654-2.087)
G	1.086(0.651-2.075)	0.619 (0.543-2.980)	1.127(1.043-3.025)	0.654 (0.598-1.601)

Group A: T1DM+CP, Group B: T1DM without CP, Group C: Chronic Periodontitis (CP), Group D: Healthy Group, T1DM: Type 1 diabetes mellitus.

When considering the genotype and clinical form of CP, the result was not statistically significant. In allelic distribution, the G allele (86.66%) was correlated with severe clinical destruction form of CP with T1DM. The odds ratio revealed that no statistical significant risk of CP was present when considering A, G alleles (IL- l7A) in patients with or without T1DM. G allele showed considerable correlation (OR=0.356) with severe form of clinical parameters in CP patients. There was no statistical difference when considering the allelic distribution (A, G) of the IL-17A among the groups (A, B, C, D). In T1DM with CP patients, there was no statistical significant difference in A, G alleles distribution when compared with the healthy group (*p*= 0.034), (*p*= 0.045) respectively (Table 4). When considering the dominant and recessive models, the patients with CP (group C) had a statistical significant difference when compared (A+G versus G) with a healthy group (*p*= 0.120) (Table 2).

**Table 4:** Distribution of the IL-17A alleles (A, G) in the groups (Chi-Square Test)

IL-17A	Group A	Group B	Group C	Group D	D vs. A (*p* Value)	D vs. B (*p* value)	D vs. C (*p* value)	A vs. B (*p* value)
A%(21.66)	13.33	16.66	33.33	23.33	0.034	0.019	0.041	0.026
G% (78.32)	86.66	83.33	66.66	76.66	0.045	0.025	0.037	0.021

*p*> 0.05. The chi-square test was used Group A: T1DM+CP, Group B: T1DM without CP, Group C: Chronic Periodontitis (CP), Group D: Healthy Group, T1DM: Type 1 diabetes mellitus.

## Discussion

Previous studies in the literature reported that IL-17A polymorphism with AA (dominant) genotype and A allele were commonly associated with type 1 and 2 diabetes and CP than GG (recessive) genotype and G allele [ 24 - 25 ]. In order to find the genotype and allele association, we evaluated IL-17A rs2275913 polymorphism in a group of patients with and without T1DM and/or CP from South Indian population.

In this study, AA, AG, and GG genotype were assessed and compared among the groups. Our results revealed that no statistical significant difference was present in genotype frequencies (*p*> 0.05) between the groups. The correlation of GG genotype in T1DM with CP and healthy group (OR=l.043, CI= 0.895-2.065) showed a higher frequency, but not statistically different when compared with T1DM without CP. In T1DM with CP patients, no statistical significant difference was found in allelic distribution of IL-17A when compared with healthy group (*p*= 0.034), (*p*= 0.045) respectively. In contrast to our finding, a study done by Linhartova *et al*. [ 19 ] showed the possible association of IL-17A (rs2275 913) AA genotype and A allele in patients of T1DM with CP. While the genotype frequencies were not statistically significant between healthy and diseased patients, GG genotype was shared higher percentage (73.3%, 66.6%, and 46.6%) of distribution among all the diseased groups. Our study also analyzed the dominant (AA, AG) and recessive genotype (GG) models in each group and compared with the healthy group (group D). T1DM with CP (group A) had a statistical significant difference when compared (AA+AG vs. GG) with healthy group (*p*= 0.068). When considering the OR, 95% CI, T1DM+CP group had no association of IL- l7A polymorphism, even though p value was significant (OR= 1.021 CI (0.654-3.543).

In CP patients, we found no statistical significant different in genotypes (AA, *p*= 0.006, AG, *p*= 0.007, GG, *p*= 0.0328) and allelic (A, *p*= 0.041, G, *p*= 0.037) distribution of IL-17A when compared with a healthy group. While the allele frequencies were not statistically significant between healthy and diseased patients, G allele was shared higher percentage (86.66%, 83.33%, and 66.66%) of the distribution among all diseased groups. This result was in accordance with the study done by Saraiva *et al*. [ 20 ] that demonstrated the GG genotype and G allele was more frequently occurred in diseased (CP, AgP) group. In contrast to our study, the in vitro study of Espinoza *et al*. [ 26 ] demonstrated healthy individuals expressing A allele of IL-17A had secreted increased amount of IL-17 cytokines than those without this allele. This could be the reason for non-significant result of our study as healthy subjects had less A (23.33%) allele when compared with G (76.66%) allele. Another study in Indian population done by Chaudhari *et al*. [ 25 ] found that IL-17A polymorphism with A allele was significantly associated with localized aggressive periodontitis and CP patients. Three Brazilian studies and one Iranian research examined the variability of IL-17A polymorphism in relation to periodontal disease and showed no association with risk of disease [ 14 , 27 ]. In contrast to this, Correa *et al*. [ 27 ] and Zacarias *et al*. [ 24 ] studies revealed the A allele and AA genotype as a risk factor for CP. In our study, no statistically significant difference was noticed in IL-17A polymorphism and periodontal status (clinical attachment loss and probing pocket depth). When considering the dominant and recessive models, the patients with CP (group C) had statistically significant difference when compared (A+G versus G) with a healthy group (*p*= 0.120). When considering Odds Ratio and 95% CI, CP group had no association of IL- I 7A poly morphism, even though p value was a significance (OR= 1.207 and CI =0.765-2.755). Interestingly, the genotype distributions of the IL- l7A variant (AA+AG vs. GG) in the group A (T1DM &CP) (*p*= 0.068) and group C (non-diabetic with CP (*p*= 0.120) were significant when compared with group B (diabetes without CP, *p*= 0.0174) patients as showed in Table 2, 3. This could be explained the possible association of IL- l7A polymorphism with CP either in the presence or absence of diabetes. This difference in finding among the South Indian population could be attributed to the genetic variation, which has seen like in European, Iranian, and Brazilian populations.

There are some limitations in this study, which are described as follows. First, the study was done with small sample size of patients included in each group. Secondly, uncontrolled diabetes patients were not included for in this study. This could lead to the genotypic and allelic variation bias in the IL-17A polymorphism. Third, the patients were selected from the same zone of location; this could mislead the genetic variation. Fourth, the diabetes and non-diabetes groups were not age-matched. However, within the limitations, the study showed no association of IL- I 7A polymorphism in T1DM with CP patients that indicates the possible importance in disease pathology of chronic periodontitis.

## Conclusion

The present study demonstrated no association of IL-17A polymorphism in CP with T1DM patients of the south Indian population. Further studies with large sample size might give an evidence-based overview of IL-17A polymorphisms and CP. In addition, there was a genotype similarity among the patients and controls; we hypothesized that IL- I 7A is not a polymorphic within our Indian population. 
